# Do Chronic Pain Patients Wish Spiritual Aspects to Be Integrated in Their Medical Treatment? A Cross-Sectional Study of Multiple Facilities

**DOI:** 10.3389/fpsyt.2021.685158

**Published:** 2021-06-17

**Authors:** Karin Hasenfratz, Hanspeter Moergeli, Haiko Sprott, André Ljutow, René Hefti, Isabelle Rittmayer, Simon Peng-Keller, Michael Rufer

**Affiliations:** ^1^Faculty of Theology, University of Zurich, Zurich, Switzerland; ^2^Department of Consultation-Liaison Psychiatry and Psychosomatic Medicine, University Hospital Zurich, Zurich, Switzerland; ^3^University of Zurich and Arztpraxis Hottingen, Zurich, Switzerland; ^4^Swiss Paraplegic Center, Nottwil, Switzerland; ^5^Research Institute for Spirituality and Health, Klinik Stiftung für Ganzheitliche Medizin (SGM), Langenthal, Switzerland; ^6^Zürcher RehaClinic, Davos, Switzerland; ^7^Department of Psychiatry, Psychotherapy and Psychosomatics, Psychiatric University Hospital Zurich, University of Zurich, Zurich, Switzerland

**Keywords:** chronic pain, spirituality, patients' needs, multimodal therapy, spiritual resources

## Abstract

**Background:** Chronic pain is a complex, multidimensional experience. Spirituality is hypothesized to impact pain experience in various ways. Nevertheless, the role that spirituality plays in multimodal pain therapy remains controversial and, to date, quantitative data on whether and for which patients spiritual aspects should be considered in the treatment of chronic pain is lacking. The aim of this study was thus to investigate the proportion and characteristics of patients with chronic pain who wish spiritual aspects to be integrated in their treatment.

**Methods:** Two hundred nine patients with chronic pain were recruited from five inpatient departments and outpatient clinics in the German-speaking part of Switzerland. Patients filled out validated questionnaires, such as the Hospital Anxiety and Depression Scale (HADS), the Resilience Scale (RS-11), the Spiritual and Religious Attitudes in Dealing with Illness (SpREUK), and the 12-item Spiritual Well-Being Scale (FACIT-Sp-12).

**Results:** More than 60% (CI_95%_: 55.5–67.9%) of the patients wanted to address spiritual aspects in their treatment. These patients were significantly younger, had higher levels of education, and suffered from more frequent and more severe pain than patients who did not wish to address spiritual aspects. Furthermore, there were high correlations with existing spiritual resources and higher scores of spirituality.

**Conclusions:** These results confirm that the majority of chronic pain patients wish spiritual aspects to be considered in their treatment. Additionally, the finding that these patients had more spiritual resources underlines the importance of integrating spiritual aspects in a resource-oriented, patient-centered care approach for this condition.

## Introduction

Chronic pain is a complex, multidimensional experience (1) and, consequently, most treatment strategies pursue an interdisciplinary, multimodal approach based on the biopsychosocial model (2, 3). Focusing the therapy on the resources available to the individual patient is becoming increasingly important (4). These resources should include spiritual resources as part of a comprehensive, patient-centered treatment approach.

Spirituality can be conceptualized as a fundamental dimension of human life. In 1984, the World Health Assembly passed a resolution which, for the first time, included the spiritual dimension as an important aspect of healthcare (5). Despite growing evidence of the role of spirituality in the experience of chronic pain, spiritual issues have a potentially underestimated value in the treatment of this condition (6).

Several studies have found significant relationships between spirituality and physical and mental health and well-being [for reviews see (7–10)]. Although spirituality was not associated exclusively with positive outcomes (9, 11), a number of studies suggested that spirituality could act as an important and useful resource for many patients coping with chronic pain (12–14). Further, this resource does not necessarily seem to be linked to religious affiliation as it has been shown that even patients who do not consider themselves to be religious or spiritual have spiritual needs (15).

A survey involving a large sample of general practitioners in Switzerland showed that physicians often felt insecure in their professional dealings with spiritual issues (16). Difficulties include fear of inadequacy in addressing these personal issues, both for the practitioner and the patient. Indeed, for some patients, the subject of “spirituality” is considered taboo (17). For physicians, the ability to take spiritual and religious aspects into account in medical practice contributes to their professional self-perception, and requires appropriate training (18). Another difficulty is the ambiguity and complexity of the concept of spirituality, which can be quite helpful in clinical practice (19) and in health policy (5), but poses problems for scientific operationalization. Especially the distinction to the concept of religion is the subject of extensive discussions^1^.

Spirituality—and the ways in which it can be appropriately discussed in relation to medical treatment—not only varies greatly from individual to individual but is also strongly influenced by cultural factors. Research from other countries and cultures or with other patient groups can therefore only be transferred to chronic pain patients in Switzerland to a limited extent. The population of Switzerland is characterized by a denominational diversity that has undergone great changes in recent decades (20). This development was the background for a research project on spiritual care in the treatment of chronic pain in Switzerland in order to better validate a possible integration of spirituality in the multimodal treatment of this condition. This project was funded by the Swiss National Research Program “Smarter Health Care” [NRP-74 (21)]. Findings from a previously published, qualitative study within this NRP-74 research project suggested that patients as well as health care professionals consider spiritual aspects to be important in the treatment of chronic pain (22, 23). Although this qualitative study yielded valuable insights regarding which aspects chronic pain patients consider to be spiritual and how they wish to address them, quantitative data on whether patients with chronic pain in Switzerland wish spiritual aspects to be taken into account in their treatment is still lacking.

Given these considerations, this multi-facility, cross-sectional study aimed to examine whether chronic pain patients wish spiritual aspects to be integrated in their treatment. Validated questionnaires of spiritual and mental health indicators were used to further investigate possible differences in patient characteristics between those who wanted and those who did not want to address spiritual aspects in their treatment.

## Methods

### Sample Recruitment

This study was part of a larger research project about the validation of a screening instrument which assess spiritual resources and spiritual distress (24). Patients were recruited from five different inpatient departments and outpatient clinics in the German-speaking part of Switzerland by their respective attending physician between November 2018 and November 2019. Before any participants were enrolled, the study was approved by the corresponding ethics review board of each institution. Inclusion criteria were: (1) aged between 18 and 80 years, (2) suffering from chronic pain, (3) willingness to participate in the study, and (4) sufficient German language skills. Exclusion criteria were: (1) an active life-threatening disease, and (2) levels of cognitive impairment that would hinder participation. Written informed consent was obtained from all study participants before completing the survey. Of the original 225 patients recruited, 10 were excluded due to incomplete answers, four chose not to return the survey, and two patients had to be excluded because they were either too young or too old. The remaining 209 patients were included, resulting in an inclusion rate of 92.9%.

### Integration of Spiritual Aspects in Treatment

The participants were given the following definition of how the term “spiritual” was to be understood: “Spiritual” refers to meaningful experiences, attitudes and practices that connect a person with what sustains and inspires his or her life. They can be religious or non-religious.” In order to accommodate cultural and ideological diversity and in accordance with WHO documents (25), we used a purposely inclusive definition. Subsequently, the participants were asked if they thought that spiritual aspects should be integrated in their treatment. No specific spiritual interventions have been mentioned. The answers on a 6-point Likert-scale ranged from “no, not at all” to “yes, certainly.” This 6-item construction allowed us to consider those who were not completely decided (e.g., “rather yes” or “rather no”) in the initial stages of analysis but, in later stages, it allowed us to split the distribution into two; those who would (rather) wish spiritual aspects to be addressed in treatment and those who would (rather) not.

### Demographic Data and Pain History

All participants completed a sociodemographic questionnaire to obtain data on their age in years, gender, in which country they had grown up, level of education, socioeconomic and marital status, as well as denominational affiliation. The participants were asked about the duration of their illness, pain frequency, intensity and localization, use of analgesics, and whether they had been granted leave from work by their attending physician.

The medical diagnoses, which were used to characterize the sample, were obtained from the attending physicians. Based on this information, the main somatic diagnoses were classified according to the subcategories of chronic pain in ICD-11: chronic primary pain (including fibromyalgia and non-specific back pain), chronic cancer pain, chronic postsurgical and posttraumatic pain, chronic neuropathic pain, chronic headache and orofacial pain, chronic visceral pain, and chronic musculoskeletal pain (26). The main psychiatric diagnoses were allocated to the following categories: depression, anxiety disorders, personality disorders, somatoform pain disorders, and others [including posttraumatic stress disorder (PTSD) and fatigue].

### Measures of Spirituality

To avoid exclusive definitions prior to any explanation or definition of spirituality, the patients were asked for a self-categorization rating of how “religious” and “spiritual” they considered themselves to be, each on a scale from 0 to 10.

To assess the spiritual resources and spiritual and religious attitudes of patients, we used the German version of the Spiritual and Religious Attitudes in Dealing with Illness (SpREUK) questionnaire (27). This 15-item questionnaire is self-rated and is often applied in health care research to assess patients' interest in spiritual and religious concerns in a secular society. It “relies on essential motifs found in counseling interviews with chronic disease patients” (28). In addition, it is not biased for or against any particular religion (29). It measures the factors “Search” (for support/access), “Trust” (in higher guidance/source), and “Reflection” (positive interpretation of disease). Each of these subscales consists of 5 items to be rated on a 5-point Likert scale ranging from 0 to 4. In each subscale, the sum of the items is transformed into a total between 0 and 100. Internal consistencies (Cronbach's alpha) of these factors were high and ranged from 0.85 (“Reflection”) to 0.92 (“Trust”).

The 12-item Spiritual Well-Being Scale (FACIT-Sp-12) is a widely used measure of spiritual well-being among people with cancer and has been demonstrated to have good validity and reliability (30). Each item is scored on a 5-point scale (total range: 0–48). Cronbach's alpha was 0.88 in the present study.

### Psychological Measures

The Distress Thermometer (DT) is recommended as a screening tool for the assessment of psychosocial distress in patients and is measured on a scale from 0 (no distress) to 10 (extreme distress) (31). The Hospital Anxiety and Depression Scale (HADS) is an often used 14-item measure for general psychological distress in patients with somatic illnesses (32, 33). Although there is insufficiently strong evidence to separate the two subscales anxiety and depression, the HADS yields a strong general factor (34). It consists of 14 items rated on a 4-point Likert scale (total range: 0–42). Internal consistency in the present study was 0.92 for the HADS total score.

In order to assess resilience, the German version of the Resilience Scale (RS-11) with 11 items was used (35). Each item was rated on a 7-point Likert scale (total range: 11–77). The Resilience Scale considers resilience as a one-dimensional, enduring personality trait that is operationalized by questions regarding “personal competence” and “acceptance of self and life” (36). Cronbach's alpha was 0.91 in the present study.

### Statistical Analysis

Study data were collected and managed using REDCap electronic data capture tools (37). Statistical analyses were performed using the Statistical Package for the Social Sciences (IBM SPSS Statistics, Version 25). Descriptive analyses were used to characterize the sample. In order to calculate the 95% confidence interval (CI_95%_) for the proportion of patients wishing to address spiritual aspects in their treatment, bootstrapping based on 1,000 bootstrap samples was performed. To assess differences between the two groups of patients (i.e., those who wished to address spiritual aspects in their treatment and those who did not), two-tailed *t*-tests for independent samples were applied to compare continuous variables, while Fisher's exact tests were used to compare the nominal data. A *P*-value < 0.05 was considered to be statistically significant. For the multivariate analysis, we modeled a backward stepwise logistic regression to determine to what extent socio-demographic, pain-related and spirituality-related variables predict the patients' wish to address spiritual aspects in treatment.

## Results

### Socio-Demographic and Clinical Sample Characteristics

Sociodemographic characteristics of the final sample consisting of 209 patients with chronic pain conditions are displayed in Table 1.

**Table 1:** Sociodemographic characteristics of patients with chronic pain (*n* = 209).

**Characteristics**	**% or mean (SD)**
**Gender**	
Male	26.8
Female	73.2
**Age in years, mean (SD)**	51.8 (15.0)
**Grown up in**	
Switzerland (CH)	84.2
Europe (except CH)	12.4
Other countries	3.3
**Marital status**	
Married/living with partner	54.3
Divorced/separated	14.4
Widowed	4.8
Single	26.4
**Education**	
University or higher vocational college	23.4
High school (university-entrance diploma)	7.2
Apprenticeship	46.9
Secondary school (mandatory school)	13.4
**Denominational affiliation**	
Roman Catholic	25.5
Protestant	28.4
Other Christian denomination	14.9
Muslim	5.3
Unaffiliated with any religion	18.3
Atheist	5.3
Other	2.4

*SD, standard deviation*.

Regarding sample proportion, 67.5% of the patients were recruited from outpatient facilities and 32.5% from inpatient facilities. The mean duration of chronic pain was 11.0 ± 11.3 years. Most of the patients suffered from primary chronic pain, including unspecific back pain and fibromyalgia (46.1%), or from secondary musculoskeletal pain (31.9%). Patients with neuropathic pain (8.8%), chronic headache and orofacial pain (7.8%), chronic postsurgical or posttraumatic pain (4.4%), and chronic secondary visceral pain (1.0%) were less frequently represented. The proportion of patients with a psychiatric diagnosis was 54.1% in total: 26.3% had a depressive disorder, 9.1% a somatoform disorder, 3.8% a personality disorder, 2.4% an anxiety or obsessive-compulsive disorder, and 12.4% other psychiatric diagnoses.

### Religious Affiliation, Religiosity, and Spirituality

While the denominational affiliation in our sample was predominantly Christian (a total of 68.8% consisting of 28.4% Protestant, 25.5% Roman Catholic and 14.9% other Christian communities), 5.3% were Muslim, and 26.0% of the participants were unaffiliated/non-denominational or defined themselves as atheist (Table 1). In self-categorization on an 11-point Likert scale, the whole sample considered themselves more spiritual (Mean = 5.05, SD = 3.09) than religious (Mean = 4.52, SD = 3.03).

There were some associations between measures of spirituality and health parameters: Spiritual well-being (FACIT-Sp) correlated strongly negative with higher levels of psychological distress (HADS, *r* = −0.56, *P* < 0.001), and slightly negative with the Distress Thermometer (*r* = −0.19, *P* < 0.01). The SpREUK subscale “Search” correlated slightly with the Distress Thermometer (*r* = 0.15, *P* < 0.05).

### Should Spiritual Aspects Be Considered in Treatment?

The answer to the question of whether spiritual aspects should be considered in treatment on the 6-point Likert scale created for this study showed a bimodal distribution with peaks at the two extremes; the majority of participants chose either 6/6 for “yes, certainly” (26.3%), or 1/6 for “no, not at all” (20.1%), while the options between the extremes were less frequently chosen (2/6 6.2%, 3/6 12.0%, 4/6 16.3%, 5/6 19.1%).

Considering the bimodal distribution, for all further analyses the answers were dichotomized into two groups with a cut-off in the middle on the 6-point Likert scale. This new grouping showed that 61.7% (CI_95%_: 55.5–67.9%) of the study participants preferred spiritual aspects to be addressed in their treatment, while 38.3% preferred spiritual not to aspects be considered in their treatment.

Those who indicated that they wished spiritual aspects to be considered in their treatment were significantly younger in age than those who did not wish to address spiritual aspects (M = 49.6, SD = 14.6 years vs. M = 55.5, SD = 15.1 years, *P* < 0.01). In addition, the former group had grown up more often in Switzerland than in other countries and were more likely to have an academic-level education. There were no significant differences concerning gender or marital status (see Table 2).

**Table 2:** Comparison of the sociodemographic characteristics of patients who want spiritual issues to be considered in their treatment (yes) with those who do not (no) (*n* = 209).

**Characteristics**	**Yes (%)**	**No (%)**	***P*-value (Fisher's exact)**
**Gender**			0.426
Male	57.1	42.9	
Female	63.4	36.6	
**Grown up in**			0.044^*^
Switzerland (CH)	65.3	34.7	
Europe (except CH)	42.3	57.7	
Other countries	42.9	57.1	
**Marital status**			0.248
Married/with partner	61.9	38.1	
Divorced/separated	53.3	46.7	
Widowed	40.0	60.0	
Single	69.1	30.9	
**Education**			<0.001^***^
University or higher vocational college	83.7	16.3	
High school	53.3	46.7	
Apprenticeship	63.3	36.7	
Secondary school	39.3	60.7	

* *p < 0.05;*

*** *p < 0.001. Bold values are significant p-values*.

### Considering Spiritual Aspects in Treatment: Relation to Disease, Psychological Distress, and Resilience

Regarding the severity of the disease, small but significant differences could be found. Specifically, the group of patients who wished to address spiritual aspects had a higher number of days with pain within the last 2 weeks, and slightly increased intensity of pain during the same period. No significant differences were found between groups for the duration of the chronic pain, frequency of intake of analgesics, or a general practitioner's permission to take sick leave (see Table 3).

**Table 3:** Comparison of the patients who want spiritual issues to be considered in their treatment (yes) with those who do not (no) regarding clinical variables (*n* = 209).

**Variables**	**Yes (% or mean, SD)**	**No (% or mean, SD)**	***P*-value**
Pain intensity on NRS 0–10, mean (SD)	5.88 (1.91)	5.21 (2.15)	**0.019**^a^^*^
Days with pain within the last 2 weeks, mean (SD)	12.07 (3.43)	10.75 (4.37)	**0.023**^a^^*^
Duration of disease in years, mean (SD)	11.53 (11.34)	10.06 (11.11)	0.369^a^
Setting %			
Out-patients	58.9	41.1	0.229^b^
In-patients	67.6	32.4	
Supposed cause for chronic pain %			
Primarily somatic	54.5	45.5	0.109^b^
Psych. comorbidity	67.0	33.0	
Primarily psychiatric	75.0	25.0	
Sick certificate % (*n* = 200)			
Yes	69.1	30.9	0.137^b^
No	58.0	42.0	
Analgesics % (*n* = 208)			
Rarely or never	59.0	41.0	0.560^b^
Frequently or daily	63.1	36.9	
HADS (mean, SD) (*n* = 208)	16.91 (7.74)	14.76 (9.37)	0.088^a^
Distress Thermometer (mean, SD)	6.24 (2.21)	5.68 (2.63)	0.111^a^
RS-11 (mean, SD) (*n* = 208)	54.73 (11.52)	54.62 (13.79)	0.951^a^

a *t-test (2-tailed)*.

b *Fisher's exact (2-tailed)*.

* *p <0.05*.

*SD, standard deviation; HADS, Hospital Anxiety and Depression Scale; RS-11, Resilience Scale. Bold values are significant p-values*.

Although there was a tendency for those who wished to address spiritual aspects to suffer from higher levels of psychological distress (HADS) attributed to the chronic pain condition compared to the other group, there were no significant differences found regarding the Distress Thermometer. There was also no correlation with resilience as measured by the RS-11 scale or the Resilience Scale RS-11 scores between groups. The RS-11 is conceptualizes Resilience as a personal characteristic which correlates with general self-efficacy (36).

### Considering Spiritual Aspects in Treatment: Relation to Parameters of Spirituality

Regarding denominational affiliation, the wish to incorporate spiritual aspects in treatment was unequally distributed (Fisher's exact, *P* < 0.01, see Table 4). However, comparing subgroups showed no significant differences between Roman Catholics and Protestants (Fisher's exact, *P* = 0.253), nor between the unaffiliated and atheists concerning the wish to include spiritual aspects in the treatment. Significant differences could only be found when comparing the small subgroup of Muslim patients with the subgroup of “other Christian denominations” or when comparing this latter group with the Protestant patients (both comparisons: Fisher's exact, *P* < 0.01).

**Table 4:** Comparison of the patients who want spiritual issues to be considered in their treatment (yes) with those who do not (no) regarding denominational affiliation (*n* = 209).

**Denominational affiliation**	**Yes (%)**	**No (%)**	***P*-value (Fisher's exact, 2-tailed)**
Roman Catholic	50.9	49.1	
Protestant	62.7	37.3	
Other Christian denominations	90.3	9.7	
Muslim	36.4	63.6	0.004^**^
Unaffiliated	57.9	42.1	
Atheist	63.6	36.4	
Other	60.0	40.0	

** *p < 0.01. Bold values are significant p-values*.

Significant differences could be found for self-categorization of spirituality; those who wished to address spiritual aspects considered themselves to be more “spiritual,” but not significantly more “religious” than those who did not wish to address spiritual aspects (see Table 5). Highly significant differences were also found between the groups concerning spiritual resources according to the SpREUK questionnaire scores. In detail, patients who wished to address spiritual aspects had significantly higher scores in all three subscales of the SpREUK questionnaire (i.e., “Search,” “Trust,” and “Reflexion”). However, no significant differences could be found between the two groups regarding spiritual well-being (FACIT-Sp).

**Table 5:** Comparison of the patients who want spiritual issues to be considered in their treatment (yes) with those who do not (no) regarding spirituality parameters (*n* = 209).

**Variables**	**Yes** ** (mean, SD)**	**No** ** (mean, SD)**	***P*-value (*t*-test, 2-tailed)**
Self-categorization religious	4.88 (3.08)	3.99 (2.97)	0.041^*^
Self-categorization spiritual	5.91 (2.78)	3.69 (3.11)	<0.001^***^
SpREUK search	58.05 (28.39)	29.64 (25.94)	<0.001^***^
SpREUK trust	61.12 (31.91)	46.61 (28.94)	0.001^**^
SpREUK reflexion	59.96 (23.10)	41.34 (24.42)	<0.001^***^
FACIT-Sp	28.21 (9.35)	26.09 (9.55)	0.115

* *p < 0.05;*

** *p < 0.01;*

*** *p < 0.001*.

*SD, standard deviation; SpREUK, Spiritual and Religious Attitudes in Dealing with Illness; FACIT-Sp, Spiritual Well-Being Scale. Bold values are significant p-values*.

### Logistic Regression Analysis

A backward stepwise logistic regression analysis was calculated to analyze the wish to include spiritual issues in chronic pain treatment (dependent variable) in a multivariate model. The planned potential predictors for consideration in this model were all socio-demographic, pain-related, and spirituality-related variables with a bivariate significance level of *P* < 0.1. Thus, the sociodemographic and pain-related variables were: age, grew up in Switzerland (vs. all other countries), academic education (vs. all other education levels), pain intensity, pain frequency, and the HADS score. To avoid multicollinearity (38) of spirituality-related variables, only denominational affiliation, the SpREUK subscale “Search,” and self-categorization “religious” were included. SpREUK “Search” showed the strongest bivariate relation with the wish to include spiritual aspects, and related moderately to denominational affiliation and self-categorization “religious.” The remaining spirituality-related variables were not included because they were highly correlated with SpREUK “Search” (*r* > 0.6).

Table 6 displays the result of the logistic regression analysis. The wish to include spiritual aspects in the treatment of chronic pain was significantly predicted by the variables younger age, grew up in Switzerland, academic education, higher pain intensity, and higher level of SpREUK “Search.” This final model predicted those who wanted spiritual aspects to be included with a sensitivity of 84% and a specificity of 63%.

**Table 6:** Logistic regression analysis for the wish to include spiritual aspects in chronic pain treatment with socio-demographic, pain-related, and spirituality-related variables as predictors.

**Variables**	**OR**	**95% CI for OR**	***df***	***P*-value**
Age	0.974	0.952–0.997	1	0.025^*^
Grown up in CH	2.939	1.167–7.399	1	0.022^*^
Academic education	4.440	1.695–11.626	1	0.002^**^
Higher pain intensity	1.257	1.055–1.498	1	0.011^*^
SpREUK search	1.030	1.018–1.043	1	<0.001^***^

* *p < 0.05;*

** *p < 0.01;*

*** *p < 0.001*.

*OR, Odds Ratio; CI, Confidence Interval; SpREUK, Spiritual and Religious Attitudes in Dealing with Illness; df, degrees of freedom*.

*Variables removed from final model because of non-significance: pain frequency, HADS, denominational affiliation, and self-categorization religious. Bold values are significant p-values*.

## Discussion

One of the main aims of this study was to evaluate whether chronic pain patients in Switzerland would be interested in the integration of spiritual aspects in their medical treatment. Of the participating chronic pain patients, 61.7% wanted spiritual aspects to be considered in their medical treatment. This is a remarkably high proportion which is in accordance with the qualitative results of the previous focus group interviews with chronic pain patients in Switzerland (22).

These findings are also in line with the results of quantitative studies of patients with chronic diseases. In the United States, Balboni and Balboni (39) found that 58–62% of the patients with cancer in their study judged it important for their nurses or physicians to consider the patients' religious and spiritual aspects as a part of cancer care. In a systematic review of 54 studies with diverse patient groups all over the world, but mostly in the United States, Best et al. (40) reported that a majority of the patients (median = 70.5%) wanted their doctors to address spiritual aspects. In Germany, Büssing et al. (13) found that 37% of patients with chronic pain wished to talk to their physicians about spiritual or religious aspects. To our knowledge, data for Switzerland exist only for patients with severe psychiatric diseases, and this data may differ from that of chronic pain patients considerably concerning spiritual attitudes. For example, Huguelet et al. (41) assessed 147 psychiatric outpatients of whom only 25% wanted to address spiritual or religious aspects in their psychiatric care. Not only cultural differences, but also different personal understandings of the concept of spirituality might account for these partly divergent results. The definition of spirituality (see above) used in this study allows for a broad interpretation, which may or may not include religious aspects. However, it refers to immanent or transcendent aspects and is not to be equated with mental-wellbeing.

Our finding that a majority of the chronic pain patients in our study wanted spiritual aspects to be taken into account in treatment underlines the need to develop concepts and structures in patient-centered care in order to adequately assess and integrate spiritual aspects in treatment. If the patient's desire for spiritual aspects to be included in treatment is so common, then the question arises as to which profession this task falls to. Further it would also be necessary to clarify how these topics can be adequately addressed and which kind of interventions in the realm of spiritual care would be effective and health-promoting (42). To further explore these questions, a follow-up project has already been initiated with psychiatric patients in Switzerland.

Patients with an academic-level education and who were younger in age were more likely to want to address spiritual aspects in the treatment. This rather surprising result might be of relevance for the clinical practice, as it shows that those with higher education and who are younger cannot be assumed to be less interested in addressing spiritual aspects. The desire to discuss spiritual topics could depend to some extent on linguistic ability, which may be reflected in the higher education of these patients. The definition of spirituality given to the patients prior to asking them if they wish to address these issues was based on a broad and non-religion-specific concept of spirituality. This could also contribute to the fact that younger and more educated patients felt addressed. This result fits with the observation that especially among young people in Switzerland the practice of spiritual practices such as meditation has increased significantly in recent years (20).

It is surprising to note that, although higher pain intensity and frequency were associated with greater interest in addressing spiritual aspects in treatment, no clear association with higher psychological distress could be found. Taking into account the findings in patients with cancer, it could be assumed that a higher psychological burden is associated with increased spirituality and spiritual practice, and thus also with a greater interest in spiritual topics in treatment (43, 44). Even though chronic pain conditions may lead to spiritual suffering, the nature of it possibly differs from that experienced in relation to life-threatening diseases. Future studies should examine differences between life-threatening and non-life-threatening conditions with regard to the patient's wish to integrate spirituality in treatment.

Self-categorization of spirituality showed an interrelation with the wish to address spiritual aspects, but no significant differences could be found between the major groups of denominational affiliation (i.e., Roman Catholics and Protestants) and the unaffiliated. These results suggest that the lack of a religious affiliation should not be interpreted as indicative of a lack of interest in spiritual aspects. In the small subgroup of Muslims, only one third of respondents wished to integrate spiritual aspects into treatment. This greater reluctance may be due to the fact that Muslims feel less well-understood with their particular spiritual concerns in a secular context.

The missing associations with general resilience (RS-11) and psychological distress (HADS and DT) could be an indication that the concept of spirituality differs fundamentally from concepts within the psychological domain, and might therefore need to be considered as a separate dimension of health and disease. However, further studies are needed to elucidate the relationships between these concepts.

The integrating logistic regression model of demographic and disease-related variables had a moderate predictive power for an individual patient wanting spiritual aspects to be addressed in the treatment. There was a strong association between the wish to include spiritual aspects in the treatment and existing spiritual resources as they are measured in the SpREUK, especially for the subscale “Search.” This subscale measures the interest in searching for meaning and purpose in life as well as for a transcendental truth (29). On the one hand, this finding suggests that patients might consider spiritual search and associated resources to be important in the treatment process. On the other hand, it might suggest that those patients who consider spiritual aspects in their treatment to be important may already have specific spiritual resources. This insight could be a noteworthy contribution toward a personalized, resource-oriented multimodal treatment of chronic pain (4). For those patients who wish to include spiritual aspects in their treatment, granting permission to talk about these aspects could itself contribute to a more comprehensive treatment. It also seems prudent to develop and test interventions aimed at reducing spiritual distress and enhancing spiritual resources.

### Limitations

It is possible that there was a selection bias of patients who are particularly interested in spiritual aspects. However, our sample was not highly religious or spiritual according to the results of self-categorization (i.e., 59% of the recruited patients not considering themselves to be “religious,” and 49% not to be “spiritual”), results which reflect those of the general population of Switzerland (20). While “other Christian denominations” were slightly overrepresented in our sample (i.e., 15% compared to 7% in Switzerland's population), the distribution of denominational affiliation approximately corresponds to those in Switzerland (20). It is important to consider that patients with insufficient knowledge of German could not be included in the survey. For these foreign-language patients, aspects of spirituality could be more diverse. Furthermore, different results may be found in patients living in other countries and cultures.

Because the study was cross-sectional, no causal relationships can be inferred from the findings.

### Conclusions

A majority (61.7%) of chronic pain patients in Switzerland found it important to consider spiritual aspects in their treatment. Those patients who wished to address spiritual aspects also had more spiritual resources such as a renewed interest in spiritual issues or an intensified search for a spiritual source to cope with illness. The results of this study point to the need to consider the psychiatric and psychotherapeutic recommendations concerning the inclusion of the spiritual dimension (45, 46) and their extension to the field of multimodal pain therapy in order to develop a resource-oriented biopsychosocial-spiritual approach for chronic pain.

## Data Availability Statement

The raw data supporting the conclusions of this article will be made available by the authors, without undue reservation.

## Ethics Statement

The studies involving human participants were reviewed and approved by Cantonal Ethics Commission, Zurich. The patients/participants provided their written informed consent to participate in this study.

## Author Contributions

SP-K, MR, HM, and KH designed the study and interpreted the data HS, AL, RH, IR, and KH recruited the participants and conducted the survey. KH and HM analyzed the data. KH wrote the manuscript. All authors read and approved the final manuscript.

## Conflict of Interest

The authors declare that the research was conducted in the absence of any commercial or financial relationships that could be construed as a potential conflict of interest.

## Footnotes

^1^Available online at: https://tif.ssrc.org/wp-content/uploads/2010/05/Why-and-How-to-Study-Spirtuality.pdf (accessed February 7, 2021).
